# The bioavailability of iron picolinate is comparable to iron sulfate when fortified into a complementary fruit yogurt: a stable iron isotope study in young women

**DOI:** 10.1007/s00394-019-01989-4

**Published:** 2019-06-11

**Authors:** Magalie Sabatier, Dominik Grathwohl, Maurice Beaumont, Karine Groulx, Laurence F. Guignard, Peter Kastenmayer, Stephane Dubascoux, Janique Richoz, Edwin Habeych, Christophe Zeder, Diego Moretti, Michael B. Zimmermann

**Affiliations:** 1 Nestlé Research, Société des Produits Nestlé SA, Vers-chez-les-Blanc, 1000 Lausanne 26, Switzerland; 2 Laboratory of Human Nutrition, Institute of Food, Nutrition and Health, ETH Zurich, 8092 Zurich, Switzerland

**Keywords:** Iron bioavailability, Stable isotopes, Iron picolinate, Complementary fruit yogurt, Women, Iron sulfate

## Abstract

**Purpose:**

A technological gap exists for the iron (Fe) fortification of difficult-to-fortify products, such as wet and acid food products containing polyphenols, with stable and bioavailable Fe. Fe picolinate, a novel food ingredient, was found to be stable over time in this type of matrix. The objective of this study was to measure the Fe bioavailability of Fe picolinate in a complementary fruit yogurt.

**Methods:**

The bioavailability of Fe picolinate was determined using stable iron isotopes in a double blind, randomized cross-over design in non-anemic Swiss women (*n* = 19; 25.1 ± 4.6 years). Fractional Fe absorption was measured from Fe picolinate (2.5 mg ^57^Fe per serving in two servings given morning and afternoon) and from Fe sulfate (2.5 mg ^54^Fe per serving in two servings given morning and afternoon) in a fortified dairy complementary food (i.e. yogurt containing fruits). Fe absorption was determined based on erythrocyte incorporation of isotopic labels 14 days after consumption of the last test meal.

**Results:**

Geometric mean (95% CI) fractional iron absorption from Fe picolinate and Fe sulfate were not significantly different: 5.2% (3.8–7.2%) and 5.3% (3.8–7.3%) (N.S.), respectively. Relative bioavailability of Fe picolinate versus Fe sulfate was 0.99 (0.85–1.15).

**Conclusion:**

Therefore, Fe picolinate is a promising compound for the fortification of difficult-to-fortify foods, to help meet Fe requirements of infants, young children and women of childbearing age.

## Introduction

Iron deficiency is a major nutritional problem worldwide today. It affects hundreds of millions of people and is especially prevalent in infants, young children and women of child bearing age [1]. Although the highest prevalence of iron deficiency in young and school-aged children is found in developing countries [2], it is also reported in industrialized areas [3]. In children, iron deficiency can have adverse effects on cognition, decrease motor activity, social attention and school performance, and increase susceptibility to infection [4].

To overcome micronutrient malnutrition, the World Health Organization [5] proposes three approaches i.e. food diversification and education, supplementation and fortification. Food fortification with iron is generally regarded as the most cost effective and sustainable long-term approach for reducing the prevalence of iron deficiency. Iron compounds used for fortification have to be carefully selected considering bioavailability and reactivity upon formulation. Available iron compounds for food fortification are classified according to their solubility. In general, the water-soluble ones are bioavailable but often cause inacceptable sensory changes in the product. Conversely, the water insoluble compounds create less sensory problems but their bioavailability is generally lower [6]. Many food products can be fortified with available forms of iron, but there is still a technological gap for the iron fortification of products having high moisture, low pH, containing polyphenols and having a long shelf life, such as shelf-stable yogurt containing fruits [7]. Iron picolinate, a novel food ingredient, is stable over time in this type of product (internal unpublished data). Fe picolinate is made from the complexation of iron with picolinic acid. Picolinic acid is an endogenous metabolite of tryptophan and an isomer of niacin (vitamin B3) and, therefore, a normal constituent of the human body and diet. The use of the parent compounds (i.e. chromium and zinc picolinate) has already been evaluated by the European Food Safety Authority (EFSA) as a source of minerals in food supplements for adults and children [8]. EFSA concluded that there was no safety concern for picolinate intakes ≤ 1.6 mg/kg body weight per day (maximal estimated combined exposure from chromium and zinc picolinate) as long as the upper limits of chromium and zinc are not exceeded. Therefore, no safety concerns are expected for its application in both young children (i.e. from 6 up to 36 months) and in the general population.

To our knowledge, the bioavailability of Fe picolinate has not been evaluated in humans. While the target population of the potential application is young children, Hurrell et al. and Harrington et al. have previously demonstrated that results obtained in adults on iron absorption can be extrapolated to them [9, 10]. Thus, the objective of this work was to determine the iron bioavailability of iron picolinate in comparison to iron sulfate from a complementary food (i.e. shelf-stable yogurt containing fruits) using stable iron isotope techniques in healthy young women. Our hypothesis was that there would be no significant difference in iron absorption from iron picolinate versus iron sulfate when fortified into this food matrix.

## Methodology and trials

### Subjects

Twenty subjects were selected from an initial screening of 59 women among the staff of the Nestlé Research Center and the Ecole Polytechnique of Lausanne. Subject inclusion criteria were healthy women, aged between 18 and 40 years old, with a weight below 65 kg and a plasma ferritin (PF) < 50 µg/L. Exclusion criteria were pregnancy or lactation, major chronic diseases or food allergies, infection in the 4 weeks before the study, smokers (> 5 cigarettes/day), alcohol consumption above 2 units a day, significant blood loss in the 6 months before the study and/or who had a significant weight loss within the 3 months before the study (10% and more). Intake of vitamin/mineral supplements in the 3 weeks before the study was not allowed. The study was conducted at the Metabolic Unit of the Clinical Development Unit, Nestlé Research Center, Lausanne, Switzerland according to the guidelines laid down in the Declaration of Helsinki. The study protocol was approved by the Commission for Ethical Research on Human Beings of Canton de Vaud, Switzerland (approval no.: 276/15 dated 1 Sept 2015) and written informed consent was obtained from all subjects. The study was registered at clinicalTrials.gov under the reference NCT02585661 (https://clinicaltrials.gov/ct2/show/NCT02585661).

### Study design

A controlled randomized, double blind cross-over design was used. One group (*n* = 10) started the study with the consumption of ^54^Fe sulfate fortified shelf stable yogurt, while the other group (*n* = 10) consumed ^57^Fe picolinate fortified shelf stable yogurt. On day 1, the first Fe labelled shelf stable yogurt was administered to the fasting subjects twice at 8:00 am and 3 pm, and they received a standardized lunch at noon. The following day, the second Fe labelled shelf stable yogurt was administered according to the same procedure. Apart from the standardized lunch, the diet was unrestricted during the study. During the baseline screening, 3 weeks before the labeled test meals were given, a venipuncture blood sample was collected to determine clinical chemistry parameters, hemoglobin (Hb), plasma ferritin and CRP (as an inflammation marker). Body weight and height were measured and subjects were asked to complete a short questionnaire about dietary habits. On the day of the first stable isotope administration (day 1) a venous blood sample was drawn under fasting conditions to measure Hb to be used for iron absorption calculation. A final venous blood sample was drawn 14 days after intake of the last test meal. The blood samples were sent to the ETH Zurich, Switzerland for the measurement of stable isotope ratios and calculation of Fe absorption.

### Isotopic labels

After an overnight fast, subjects consumed either the intrinsically labeled ^57^ferrous picolinate or ^54^ferrous sulfate mixed into the shelf-stable yogurt. Isotopically labeled ^57^Fe picolinate and ^54^Fe sulfate were prepared by Dr. Paul Lohmann GmbH (Emmerthal, Germany) and Innophos (NJ, USA), respectively, from isotopically enriched elemental iron (Isoflex, CA, USA). Compounds were prepared using a downscaled procedure that follows closely the process employed for the production of commercially available products. Isotopic enrichments of ^57^Fe picolinate and of ^54^Fe sulfate were 94.5% and 99.6%, respectively. Iron concentration in the labeled ^57^Fe picolinate and in the unlabeled compound was determined after mineralization in a CEM Microwave digestion system using HNO_3_/H2O_2_ according to official method EN 13805:2014 [11].

^54^Fe sulfate and unlabeled commercial Fe sulfate dried were dissolved in 0.5 M HNO_3_. Fe in digests and dissolved samples was analyzed by ICP-AES according to the Association of Official Analytical Chemists (AOAC) official method 2011.14. Solubility of the ^57^Fe picolinate, ^54^Fe sulfate as well as of unlabeled reference compound was measured at pH 1.0 as proposed by Lynch et al. [12]. Aliquots of Fe compounds containing 20 mg Fe were weighed into 500-mL conical flasks, and 250 mL of 0.1 M HCl warmed to 37 °C, were added. Flasks were placed in a shaking water bath at 37 °C and gently shaken at a rate of 1 Hz for 30 min. Aliquots of 2 mL were taken and centrifuged for 2 min at 1000*g*. Iron was analyzed in supernatants by inductively coupled plasma atomic emission spectrometry (ICP-AES) (Varian Vista MPX, Varian AG, Mulgrave, Australia) as described above.

### Test meal and stable isotope administration

Test meals consisted of a shelf stable yogurt (Jogolino^®^ strawberry, Nestlé) specifically developed for children aged from 6 months up to 3 years old. The serving size was 100 g of product containing 3 g of protein, 3.4 g of fat, 15.7 g of carbohydrate, 3.5 g strawberry puree, 150 mg of calcium, fortified with 30 mg of magnesium and 1.5 mg of zinc as indicated on the label. Isotopically labeled Fe (2.50 mg per serving) were added in powder form to the yogurt 30–60 min before consumption by the subjects. Compounds were pre-weighed in individual portions into pre-weighed glass vials with Teflon caps at an accuracy of ± 0.05 mg. Administered doses varied within ± 5% (1SD) between individuals. The shelf-stable yogurt fortified with one labeled Fe compound was consumed with 200 mL low mineralized water containing less than 130 mg/L Total Dissolved Solid (TDS). Part of the water was used to rinse the yogurt container, i.e. three times with 15 mL of water, and subjects consumed the rinse water; they were not allowed to drink or eat for 3 h following the test meals. A standardized meal was provided at noon consisting of pasta (200 g) served with tomato sauce (30 g), a green salad (50 g) served with a balsamic sauce (30 g) and a sorbet (100 g), served with mineral water. Afterwards, subjects were asked to fast for 3 h. Then, they received a second serving of the product fortified with the same label as in the morning meal using identical procedures.

### Blood collection and analysis

Blood samples were drawn by experienced nurses using EDTA-coated vacutainers (monovettes^®^, Sarstedt GmbH, Nümbrecht, Germany). Hb was measured using an automated Counter (ACT 5 diff counter, Beckman Coulter International S.A., Nyon, Switzerland). A control material of 3 levels (Coulter AC.T5 diff Control Plus) was analyzed with each set of measurements of Hb. Plasma was separated for ferritin and CRP measures by centrifugation (Sorvall RC6 + centrifuge, ThermoFisher Scientific, Osterode, Germany). Ferritin and CRP were measured with the Siemens Dimension^®^ Clinical Chemistry system using Flex^®^ reagent cartridges. Ferritin was measured using the enzyme immunoassay method and CRP using the C-Reactive Protein Extended Range (RCRP) method based on a particle enhanced turbidimetric immunoassay (PETIA) technique. Two level serum control material (Liquid Assayed Multiqual Premium) were analyzed with each ferritin and CRP measurements.

### Isotopic analysis of the blood samples

Each isotopically enriched blood sample was analyzed in duplicate for its isotopic composition. Whole blood was mineralized by microwave digestion, and Fe was separated by anion exchange chromatography and a subsequent precipitation step with ammonium hydroxide [13]. Fe isotope ratios were determined by an MC-ICP-MS instrument (Neptune; Thermo Finnigan).

### Calculation of iron absorption

Fractional iron absorption was calculated based on the incorporation of enriched ^57^Fe and ^54^Fe from absorbed isotopically labeled Fe compounds into red blood cells. Amounts of ^57^Fe and ^54^Fe isotopic label present in the blood 14 days after test meal administration were calculated based on measured shifts in the iron isotope ratios in the blood samples compared to natural iron abundancy (i.e. baseline) and the amount of iron circulating in the body. The natural iron abundancy was borrowed from a historical control of comparable women at ETH Zurich. Calculations were based on isotope dilution principles as previously described [14]. Fractional absorption expressed as percentage of dose was calculated by dividing the amount of absorbed iron label by the administered dose multiplied by 100. Circulating iron was calculated based on blood volume and hemoglobin concentration [15]. Blood volume calculations were based on height and weight [16]. For the calculation of fractional iron absorption, 80% incorporation of the absorbed iron into red blood cells was assumed [17].

### Food analysis

Fe and calcium content of the shelf-stable yogurt and the water were determined by ICP-AES after mineralization as described above.

## Statistics

The sample size calculation was based on data from a previous trial [18]. The within subject standard deviation of fractional absorption and the relative bioavailability was approximately 2.5% and 25%, respectively (estimated by the mixed model). Therefore, with eighteen subjects, the fractional iron absorption and the relative bioavailability can be measured with approx. 25%/18^1/2^ = 6% standard error which was considered as sufficiently precise. Anticipating a potential dropout of two subjects, the sample size was increased to 20. Fractional iron absorption was approximately log-normally distributed. The natural logarithm was used for the transformation. Descriptive statistics of fractional iron absorption from both compounds was displayed by geometric means and by the back transformed means ± standard deviations. Log-transformed fractional absorption was analyzed by a mixed model. Fixed-effect was compound (Fe-picolinate or Fe-sulfate), random-effect was subject. The model-based geometric mean is the exponent of the model based estimate of the predicted mean. The relative bioavailability is the exponent of the model-based treatment difference. To draw conclusions on bio-equivalence, criteria provided by FDA were used [19].

## Results

### Iron compounds and in vitro solubility

Iron contents of ^54^Fe sulfate and ^57^Fe picolinate were 28.4 and 19.3%, respectively. The experiments conducted at pH = 1 after 30 min showed similar solubility between labelled and unlabeled compounds i.e. 94.9 ± 1.4% for ^54^Fe sulfate, 96.9 ± 6.6% for commercial Fe sulfate, 97.8 ± 4.6% for ^57^Fe picolinate and 90.7 ± 0.6% for unlabeled Fe picolinate.

### Subject characteristics

Age, anthropometric and baseline characteristics for Hb and plasma ferritin for the twenty enrolled subjects are shown in Table 1. None of the subjects were anemic. Only three subjects had a plasma ferritin concentration below or at borderline of the cut-off value of 15 µg/L that defines iron deficiencies [5]. The mean (± SD) serum ferritin of the subjects was 29.9 ± 9.8 µg/L. This represents adequate but still low iron stores. At screening, all subjects had a CRP value < 4 mg/L. Plasma ferritin and Hb were not different at screening and on the first day of stable isotope administration. The analysis of the questionnaire about dietary habits revealed that none of the subjects was vegan or vegetarian. Nineteen subjects completed the study. One subject had an accident during the study (i.e. twisted ankle) not related to the study product or study procedures and did not provide the last blood sample.

**Table 1:** Baseline subject characteristics

Characteristics	Summary value (*n* = 20)		
	Mean	SD	Min–Max
Age (years)	25.1	4.6	20–30
Weight (kg)	55.8	4.4	49–62
Height (cm)	164.8	4.4	157–174
BMI (kg/m^2^)	20.5	1.4	18.7–23.7
Hb (g/L)	132.3	7.3	120.0–147.0
PF (µg/L)	29.9	9.8	10.8–50.9

Hemoglobin and plasma ferritin were measured on the morning of the day of the first stable isotope administration

*PF* plasma ferritin

### Food analysis

The results of mineral analysis showed that the quantity of iron and calcium brought by the shelf-stable yogurt was < 0.05 and 159 ± 1.9 mg/serving, respectively. The quantity of iron and calcium present in the mineral water consumed on the test day was below the limit of detection and 1.2 ± 0.1 mg/serving, respectively.

### Fe absorption

**Fig. 1:**
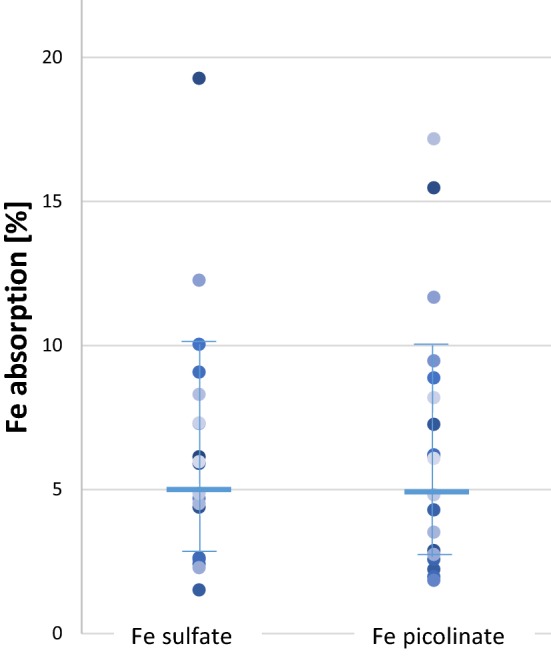
Fractional iron comparison for Fe sulfate and Fe picolinate from an iron fortified shelf-stable yogurt containing 3.5% fruits (i.e. strawberries). Values are individual data points with the horizontal bar representing the geometric mean ± SD

The geometric mean fractional iron absorption from the ^54^Fe sulfate and ^57^Fe picolinate are presented in Fig. 1. The geometric mean fractional iron absorption from the shelf stable strawberry yogurt fortified with ^57^Fe picolinate and ^54^Fe sulfate was 5.2% (95% CI 3.8–7.2%) and 5.3% (95% CI 3.8–7.3%). The relative iron absorption (RBV) from Fe picolinate in this study was 99% (95% CI 85.2–115.0) of ^54^Fe sulfate. This value is within the boundaries for bio-equivalence according to FDA: (90% CI 0.80 and 0.125) [19].

**Fig. 2:**
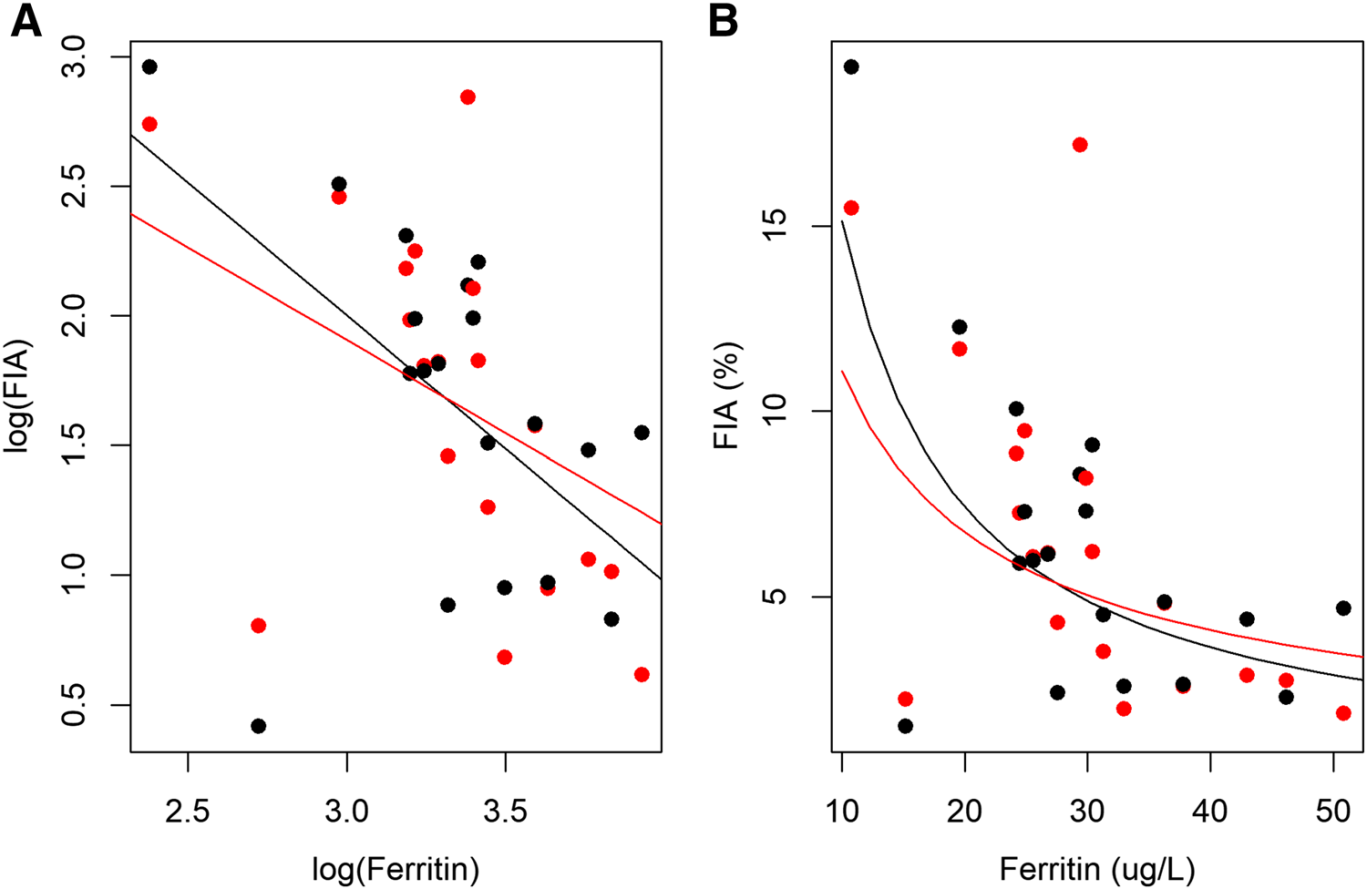
Scatterplot of fractional iron absorption values (FIA) over plasma ferritin concentration with regression lines. The regression lines are derived from the model: salt + log (ferritin) + salt × log(ferritin). **a** On a logarithmic scale, **b** on the original scale. Black dots/line FeSO_4_ and red dots/line Fe-picolinate. Slopes were not significantly different (*p* = 0.548)

In the mixed models, a time effect (day 1 versus day 2) and the effect of plasma ferritin on iron absorption was investigated. The time effect was significant (*p* = 0.04) and the point estimate was 0.84 indicating the fractional absorption on day 2 was 0.84 times less than on day 1. Absorption values and RBV corrected for this time effect were 5.2% (3.8–7.1%), 5.4% (3.8–7.3%) and 96% (84–110.5%) for ^57^Fe picolinate, ^54^Fe sulfate and the RBV, respectively, and were not significantly different than the time-effect unadjusted values. Log-plasma ferritin was also significant (*p* = 0.03) (Fig. 2) and the point estimate was 0.418 suggesting that as plasma ferritin increases from 10 to 27 µg/L, fractional iron absorption falls from 7 to 2.9%. Individual iron absorption values were corrected to a serum ferritin concentration of 30 μg/L according to Cook [20], to allow comparison to published data. This led to a geometric mean (95% CI) fractional absorption from Fe sulfate and Fe picolinate of 5.0% (3.67–6.37) and 4.8% (3.67–6.37) (N.S.), respectively.

## Discussion

This study represents the first published evaluation of iron absorption from iron picolinate in humans. When added to a shelf-stable yogurt containing fruits, iron absorption from iron picolinate was not significantly different than iron absorption from iron sulfate. This observation is in agreement with findings from the in vitro Caco-2 cell model coupled with simulated digestion (internal, unpublished data). Other picolinate salts (zinc picolinate or chromium picolinate) have been shown to be well absorbed in humans or in rats. The comparative oral absorption of zinc picolinate and zinc gluconate, an organic salt having similar bioavailability than zinc sulfate, was studied in healthy human volunteers. At the end of 4 weeks supplementation periods hair, urine and erythrocyte zinc levels was found significantly increased compared to the placebo treatment, without significant difference between zinc picolinate and gluconate [21]. The oral bioavailability of chromium from picolinate and chromium chloride was evaluated in rats using radiolabelling. The absorption of chromium picolinate was twice as high as from chromium chloride. However, 1–3 days after administration, the relative distribution of ^51^Cr from both compounds was similar in all tissues, indicating that both compounds contribute to the same storage pool and that the bioavailability (defined as retention) of chromium picolinate in rats was comparable to that chromium chloride [22]. The RBV of iron picolinate compared to ferrous sulfate was 0.99 (90% CI 0.85–1.15) in our meal matrix containing calcium and polyphenols, which is within the boundaries for bio-equivalence according to FDA [19]: (90% CI 0.80, 0.125). The RBV of iron pyrophosphate, currently used for iron fortification of this type of product, was reported to be to be 0.33 from a full cream milk powder containing ascorbic acid [18].

In the present study, the iron content and the solubility of the iron compounds were compared to ensure that both labelled and unlabeled iron compounds were equivalent. The iron contents of labelled salts were consistent with specifications for the commercial iron sulfate (i.e. 31.1%) and for the large-scale production of iron picolinate (i.e. 18.3–22.3%) provided by the supplier Dr. Paul Lohmann GmbH KG and Innophos, respectively. The solubility of the intrinsically labelled compounds were also similar to unlabeled ones, confirming their comparable physical properties. Therefore, the measured iron absorption in the present study is likely to reflect the iron absorption from the commercial compounds. The bioavailability of iron salts is partly related to their dissolution at acidic pH, and the solubility of iron picolinate and iron sulfate as measured with the standards developed by Lynch et al. [12] showed similar rates of dissolution for the two compounds. However, although in vitro solubility/dialyzability methods correlate with human absorption studies in ranking iron availability from different meals, they cannot predict iron absorption levels in humans [23].

When evaluating the absorption of iron compound in comparison to iron sulfate, to allow spotting difference that would be due to the solubility, it is preferred to enrolled subjects with low iron status. Iron absorption depends mainly on the latter. An inverse correlation between iron status and iron absorption, can be described mathematically with the use of ferritin as an indicator of iron status [24]. In a setting with subjects having high iron requirements, the lower is the solubility of the tested iron compound in comparison to iron sulfate, the higher would be the difference in iron absorption between the two salts [25]. The iron absorption from iron picolinate has been evaluated in subjects with adequate, but still low iron stores (i.e. average 29.9 μg/L). As depicted on Fig. 2 representing the fractional iron absorption values over plasma ferritin concentration, a small but not significant difference between the slopes of the regression lines could be detected. Considering that the solubility of iron picolinate was found to be similar to that of iron sulfate, differences in iron absorption between these two salts would not be expected for a population with an average serum ferritin below 20 µg/L.

The geometric mean iron absorption for ferrous sulfate from the shelf-stable yogurt (without ascorbic acid) was 5.0% after normalization to a plasma ferritin concentration of 30 µg/L. This value is comparable to those reported for composite meals. For example, Layrisse et al. reported fractional iron absorption in the range 3–14.9% from a breakfast consisting of 100 g white wheat flour, 50 g cheese and 10 g margarine, fortified with 3 mg iron [26], and Rossander et al. reported fractional iron absorption in the range of 3.7–7.8% from a breakfast fortified with 2.8–4.2 mg iron and consisting of coffee or tea, wheat rolls made from 40 g unfortified white wheat flour, 10 g margarine and either orange marmalade or 15 g cheese [27]. Nevertheless, the fractional iron absorption from ferrous sulfate in our study is significantly lower than from fresh cheese consumed alone (23.4%) (iron absorption adjusted to a serum ferritin of 30 μg/L) [28]. A part of the explanation could be the higher concentration of calcium in the shelf stable yogurt (i.e. 156 mg per serving) when compared to the fresh cheese (i.e. 70 mg per serving). Calcium inhibits iron absorption in single meal studies; however, calcium is considered a low-level inhibitor when compared to phytic acid and phenolic compounds [29]. Despite the relatively high calcium content in iron-fortified milk products, they have been shown to improve iron status in efficacy studies [30–34]. The phytate and polyphenol content in the shelf stable yogurt containing 3.5% strawberries used in our study was not measured. Although the phytate content of strawberries is negligible, the concentration of polyphenols in strawberries puree is variable and may decrease during processing (http://phenol-explorer.eu/food-processing/foods). Thus, polyphenols content of the yogurt would be relatively low compared to doses reported to impair iron absorption [35–40]. It should be noted that not only the polyphenol concentration but also the type of polyphenols present in a food matrix can affect iron absorption, and this has not been widely investigated in humans. In addition, although in single meal studies polyphenols decrease iron absorption, longer term intake studies show less of an inhibiting effect of polyphenols on Fe absorption [41]. Alternatively, the low rate of iron absorption from the yogurt might be due to its protein content. Few studies have shown the impact of casein and whey on iron absorption [42, 43], but caution might need to be used when considering these results as they were generated with semisynthetic meals and low number of subjects. Finally, it is important to remind that the present study has been performed without the addition of ascorbic acid in the yogurt. Ascorbic acid enhances iron absorption in a dose-dependent manner and is thought to exert its enhancing effect by reducing ferric to ferrous iron and by binding iron in a soluble form available for absorption [44]. Depending on the molar ratio of ascorbic acid to iron it can raise iron absorption from iron fortified milk-based food by two- to fourfold [29, 44, 45].

Young children and toddlers are particularly vulnerable to iron deficiency due to their high iron requirements to meet the needs for rapid growth and development [46]. This is particularly true after 6 months of age, due to the decreased iron liver stocks constituted during the gestational phase. Thus, when time of food diversification arrives, appropriate iron-rich food need to be introduced in their diet [47]. Many young children do not consume large quantities of foods rich in bioavailable iron, such as red meat. In addition, theoretical dietary models show that it is difficult to reach the recommended intakes of iron with a diet following available food guides for infants and young children [48]. Iron fortification of complementary food can be an effective mean to reduce the risk of iron deficiency and iron deficiency anemia [29]. Nevertheless, iron added in this type of product must be provided at adequate quantity and must be bioavailable. Our study was performed in young women with low iron stores, and suggests that iron picolinate is as bioavailable as iron sulfate from a complementary yogurt. A higher fractional absorption of iron picolinate would be likely if co-ingested with ascorbic acid by iron deficient children. In conclusion, iron picolinate could be a promising compound for the fortification of difficult-to-fortify foods, to help meet Fe requirements of infants, young children and women of childbearing age.

## References

[1] McLean E, Cogswell M, Egli I (2009). Worldwide prevalence of anaemia, WHO vitamin and mineral nutrition information system, 1993–2005. Public Health Nutr.

[2] Best C, Neufingerl N, van Geel L (2010). The nutritional status of school-aged children: why should we care?. Food Nutr Bull.

[3] Eussen S, Alles M, Uijterschout L (2015). Iron intake and status of children aged 6–36 months in Europe: a systematic review. Ann Nutr Metab.

[4] Doom JR, Georgieff MK (2014). Striking while the iron is hot: understanding the biological and neurodevelopmental effects of iron deficiency to optimize intervention in early childhood. Curr Pediatr Rep.

[5] World Health Organization (2006). Guidelines on food fortification with micronutrients. Joint WHO/FAO publication.

[6] Hurrell RF, Egli I, Kramer K, Zimmermann MB (2007). Optimizing the bioavailability of iron compounds for food fortification. Nutritional anemia.

[7] Habeych E, Van Kogelenberg V, Sagalowicz L (2016). Strategies to limit colour changes when fortifying food products with iron. Food Res Inter.

[8] European Food Safety Authority (2009). Scientific opinion on chromium picolinate, zinc picolinate and zinc picolinate dehydrate added for nutritional purposes in food supplements. EFSA J.

[9] Hurrell RF, Davidsson L, Reddy MA (1998). Comparison of iron absorption in adults and infants consuming identical infant formulas. Br J Nutr.

[10] Harrington M, Hotz C, Zeder C (2011). A comparison of the bioavailability of ferrous fumarate and ferrous sulfate in non-anemic Mexican women and children consuming a sweetened maize and milk drink. Eur J Clin Nutr.

[11] Committee AW/275. BS EN 13805:2014: Foodstuffs. Determination of trace elements. Pressure digestion. BSI (2014)

[12] Lynch RS, Bothwell T (2007). The SUSTAIN task force on iron powders. A comparison of physical properties, screening procedures and a human efficacy trial for predicting the bioavailability of the commercial elemental iron powders used for food fortification. Int J Vitam Nutr Res.

[13] Hotz K, Krayenbuehl PA, Walczyk T (2012). Mobilization of storage iron is reflected in the iron isotopic composition of blood in humans. J Biol Inorg Chem.

[14] Walczyk T, Davidsson L, Zavaleta N (1997). Stable isotope labels as a tool to determine iron absorption by Peruvian school children from a breakfast meal. Fresenius J Anal Chem.

[15] Kastenmayer P, Davidsson L, Galan P (1994). A double stable isotope technique for measuring iron absorption in infants. Br J Nutr.

[16] Brown E, Hopper J, Hodges JL (1962). Red cell, plasma and blood volume in healthy women measured by radio chromium cell-labeling and hematocrit. J Clin Invest.

[17] Hosain F, Marsaglia G, Finch CA (1967). Blood ferrokinetics in normal man. J Clin Invest.

[18] Walczyk T, Kastenmayer P, Bonsmann SSG (2013). Ferrous ammonium phosphate (FeNH4PO4) as a new food fortificant: iron bioavailability compared to ferrous sulfate and ferric pyrophosphate from an instant milk drink. Eur J Nutr.

[19] Food and Drug Administration (FDA) (2013) Guidance for industry. Bioequivalence studies with pharmacokinetic endpoints for drugs. https://www.fda.gov/Drugs/GuidanceComplianceRegulatoryInformation/Guidances/default.htm

[20] Cook JD, Dassenko SA, Lynch SR (1991). Assessment of the role of nonheme-iron availability in iron balance. Am J Clin Nutr.

[21] Barrie SA, Wright JV, Pizzorno JE (1987). Comparative absorption of zinc picolinate, zinc citrate and zinc gluconate in humans. Agents Actions.

[22] Kottwitz K, Laschinsky N, Fischer R (2009). Absorption, excretion and retention of 51Cr from labelled Cr-(III)-picolinate in rats. Biometals.

[23] Sandberg AS (2005). Methods and options for in vitro dialyzability; benefits and limitations. Int J Vitam Nutr Res.

[24] Hurrell RF, Egli I (2010). Iron bioavailability and dietary reference values. Am J Clin Nutr.

[25] Moretti D, Zimmermann MB, Wegmüller R (2006). Iron status and food matrix strongly affect the relative bioavailability of ferric pyrophosphate in humans. Am J Clin Nutr.

[26] Layrisse M, García-Casal MN, Solano L (2000). Iron bioavailability in humans from breakfasts enriched with iron bis-glycine chelate, phytates and polyphenols. J Nutr.

[27] Rossander L, Hallberg L, Björn-Rasmussen E (1979). Absorption of iron from breakfast meals. Am J Clin Nutr.

[28] Sabatier M, Egli I, Hurrell R (2016). Iron bioavailability from fresh cheese fortified with iron-enriched yeast. Eur J Nutr.

[29] Walczyk T, Muthayya S, Wegmüller R (2014). Inhibition of iron absorption by calcium is modest in an iron-fortified, casein- and whey-based drink in Indian children and is easily compensated for by addition of ascorbic acid. J Nutr.

[30] Sazawal S, Ahsan Habib AKM, Dhingra U (2013). Impact of micronutrient fortification of yoghurt on micronutrient status markers and growth—a randomized double blind controlled trial among school children in Bangladesh. BMC Public Health.

[31] Eichler K, Wieser S, Rüthemann I (2012). Effects of micronutrient fortified milk and cereal food for infants and children: a systematic review. BMC Public Health.

[32] Mauleñn I, Villagomez S, Soler E (1999). Impacto nutricio del consumo de una leche entera adicionada con vitaminas y minerales en ninos. Salud Publica Mex.

[33] Neumann CG, Bwibo NO, Murphy SP (2003). Animal source foods improve dietary quality, micronutrient status, growth and cognitive function in Kenyan school children: background, study design and baseline findings. J Nutr.

[34] Whaley SE, Signam M, Neumann C (2003). The impact of dietary intervention on the cognitive development of Kenyan school children. J Nutr.

[35] Brune M, Rossander L, Hallberg L (1989). Iron absorption and phenolic compounds: importance of different phenolic structures. Eur J Clin Nutr.

[36] Cook JD, Reddy MB, Hurrell RF (1995). The effect of red and white wines on nonheme-iron absorption in humans. Am J Clin Nutr.

[37] Hurrell RF, Reddy MB, Cook JD (1999). Inhibition of non-haem iron absorption in man by polyphenolic-containing beverages. Br J Nutr.

[38] Samman S, Sandstrom B, Toft MB (2001). Green tea or rosemary extract added to foods reduces nonheme-iron absorption. Am J Clin Nutr.

[39] Lesjak M, Hoque R, Balesaria S (2014). Quercetin inhibits intestinal iron absorption and ferroportin transporter expression in vivo and in vitro. PLoS One.

[40] Petry N, Egli I, Zeder C (2010). Polyphenols and phytic acid contribute to the low iron bioavailability from common beans in young women. J Nutr.

[41] Beck KL, Conlon CA, Kruger R (2014). Dietary determinants of and possible solutions to iron deficiency for young women living in industrialized countries: a review. Nutrients.

[42] Cook JD, Morck TA, Lynch SR (1981). The inhibitory effect of soy products on nonheme iron absorption in man. Am J Clin Nutr.

[43] Hurrell RF, Lynch SR, Trinidad TP (1989). Iron absorption in humans as influenced by bovine milk proteins. Am J Clin Nutr.

[44] Teucher B, Olivares M, Cori H (2004). Enhancers of iron absorption: ascorbic acid and other organic acids. Int J Vitam Nutr Res.

[45] Pauline M, Verghese ST, Srinivasu BY (2018). Effect of ascorbic acid rich, micro-nutrient fortified supplement on the iron bioavailability of ferric pyrophosphate from a milk based beverage in Indian school children. Asia Pac J Clin Nutr.

[46] Krebs NF (2001). Bioavailability of dietary supplements and impact of physiologic state: infants, children and adolescents. J Nutr.

[47] Fewtrell M, Bronsky J, Campoy C (2017). Complementary feeding: a position paper by the European Society for Paediatric Gastroenterology, Hepatology, and Nutrition (ESPGHAN) Committee on Nutrition. J Pediatr Gastroenterol Nutr.

[48] Ferguson EL, Darmon N, Fahmida U (2006). Design of optimal food-based complementary feeding recommendations and identification of key “problem nutrients” using goal programming. J Nutr.

